# Special Issue “Cancer Biomarker: Current Status and Future Perspectives”

**DOI:** 10.3390/ijms26052164

**Published:** 2025-02-28

**Authors:** Susan Costantini

**Affiliations:** Experimental Pharmacology Unit, Istituto Nazionale Tumori-IRCCS-Fondazione G. Pascale, 80131 Naples, Italy; s.costantini@istitutotumori.na.it; Tel.: +39-0825-1911729

In recent years, advancements in omics technologies have significantly accelerated the identification of a broad spectrum of biomarkers based on DNA, RNA, microRNAs (miRNAs), and long non-coding RNAs, as well as proteins and metabolic and lipid alterations (Figure 1) [1]. These biomarkers, when integrated with clinical and pathological data, provide valuable insights into cancer progression, prognosis, recurrence, and responses to treatment [2]. For instance, the discovery of mutations in the KRAS gene and overexpression of HER2 have provided robust prognostic markers for predicting disease progression and treatment response [3,4]. Similarly, miRNA profiles, such as the upregulation of miR-21 in various cancers, has shown promise as a non-invasive diagnostic and prognostic biomarker [5]. In addition to genetic and RNA-based biomarkers, metabolites have emerged as key players in cancer diagnostics. For example, elevated levels of lactate and pyruvate, often associated with the Warburg effect, have been identified as biomarkers for several cancer types, including glioblastoma and breast cancer, reflecting the tumor’s altered metabolism [6]. Furthermore, changes in lipid profiles, such as the upregulation of phosphatidylcholines and sphingolipids in ovarian cancer, are being explored as potential biomarkers for early detection and monitoring of the treatment response [7].

On the other hand, the advent of “liquid biopsies” has revolutionized cancer diagnostics by providing a non-invasive alternative to traditional tissue biopsies. Liquid biopsies, typically involving the analysis of cell-free (cf)DNA, exosomes, circulating tumor cells (CTCs), and various molecular components such as metabolites, lipids, and microRNAs (miRNAs), enable real-time monitoring of the patient’s pathophysiological state. This approach offers critical insights into the temporal and spatial clonal evolution of tumors, revealing key molecular alterations that reflect the dynamics of cancer progression [8].

In this context, this Special Issue “Cancer Biomarker: Current Status and Future Perspectives” provides a collection of six papers comprising a case report, two reviews, and three research articles, aimed to present recent research on the identification of novel genetic, epigenetic, protein, and metabolic cancer biomarkers.

In the case report, Nolano et al. (2023) described germline variants in Ataxia-telangiectasia mutated (ATM) and MutL protein homolog (MLH) 1 genes in a 16-year-old boy who developed a precancerous colonic lesion and had a clinical suspicion of Lynch Syndrome (LS) [9]. The latter is an autosomal dominant inherited disorder, associated with pathogenic variants in DNA mismatch repair (MMR) genes such as ATM, MLH1, MutS homolog (MSH) 1 and 6, and postmeiotic segregation increased (PMS) 2 [10]. LS predisposes mainly to endometrial and colorectal cancer, but also, if in a lower percentage, to bladder, pancreatic, prostate, small intestine, and stomach cancer [11]. The authors reported that after colonoscopy for rectal bleeding, the patient had a rectal adenomatous polyp removed. The histological diagnosis was tubular adenoma with low-grade glandular dysplasia with chronic inflammatory lymphocytic infiltration. Since the proband’s family history was positive for adenomatous polyps and cancers, microsatellite instability (MSI) was analyzed by detecting high MSI status in tissue DNA. Subsequently, the analysis of MLH1 and MSH2 exons by Sanger sequencing evidenced a deletion of the four intronic bases (GTTT) at the level of intron 7 in MLH1, named c.589-9_589-6delGTTT. This mutation was classified as a likely pathogenic variant in the international database of the InSiGHT Group (http://www.insight-group.org/, accessed on 5 March 2023). Through “in silico” analysis, the authors demonstrated that this variant could change the canonical splicing acceptor site, activating a new cryptic splicing site. This finding was confirmed by PCR analysis, which revealed a splicing isoform of MLH1 mRNA resulting from the skipping of exon 8. The presence of the c.589-9_589-6delGTTT variant in the MLH1 gene was also confirmed by next-generation sequencing (NGS). Moreover, this analysis identified also two variants in the ATM gene, c.5975A>C p.(Lys1992Thr) and c.8734A>G p.(Arg2912Gly), classified as being of uncertain significance and probably pathogenic, respectively, according to American College of Medical Genetics and Genomics criteria. Nolano et al.’s (2023) conclusion underlined that the phenotype of their case was the result of a synergistic effect between the identified variants, suggesting that it is a challenge for researchers to better understand how risk alleles in various colorectal cancer-prone genes interact in increasing the probability that a LS carrier can develop cancer [9].

In the first review, Owe-Larsson et al. (2023) focused their attention on cocaine- and amphetamine-regulated transcript (CART) neuropeptide, encoded by the CARTPT gene, and its implications in cancer [12]. The functions of the CART neuropeptide range from modulating behavior, stress, and neuropathic pain [13], inhibiting food intake by acting as a satiety signal [14], developing drug-dependency [15], and acting as a strong antioxidant by neutralizing reactive oxygen species (ROS) [16]. To provide a description of the current research related to CART’s role in cancer, in accordance with the preferred reporting items for systematic review and meta-analysis (PRISMA) 2020 statement guidelines, the authors searched for articles in English, published from 2000 to 2022 in Medline, PubMed, Scopus, and Web of Science databases, using the following keywords, “Cocaine- and amphetamine-regulated transcript” OR “Cocaine and amphetamine regulated transcript” AND “cancer*” OR “malignan*” OR “neoplas*” OR “growth factor*” OR “GPR160”, in combinations with and without “CART”. After removing duplicates, reviews, case reports, book chapters, research on animals, and irrelevant outcomes, the authors selected nineteen full-text publications among which (i) four papers described signaling pathways which were shown to be activated by CART; (ii) four papers reported studies on cell lines; and (iii) five, one, and five papers investigated CART’s role in breast cancer, glioma, and neuroendocrine tumors, respectively. This research evidenced that CARTPT acts as an oncogene, activating the ERK pathway [17], inhibiting apoptosis [18], increasing levels of cyclin D1 [19], and protecting breast cancer cells from tamoxifen-induced cell death [20]. In their conclusion, Owe-Larsson et al. (2023) suggested that CART represents a potential diagnostic biomarker in various cancers by leading to the improvement of early cancer detection, and its potential role in the modulation of neoplastic processes is evident and demonstrated in the literature. Thus, the identification of precise mechanisms of CART action may be useful to design novel anti-cancer agents and new therapeutic approaches [12].

In the second review, Holmannova et al. (2024) focused on the most significant changes at the genomic level (DNA damage, epigenetic changes, and telomere shortening) and non-genomic changes, indicated as hallmarks of aging [21]. Many mechanisms based on theories describing aging operate also during carcinogenesis [22]. Understanding the common non-genomic hallmarks of aging and cancer, and how they are correlated between them, will permit the development of approaches that could influence these hallmarks by slowing aging and also preventing cancer. Since many studies focused on genomic hallmarks, even if non-genomic hallmarks are important because they may cause genomic damage and increase the expression of genomic hallmarks, this review is organized into seven chapters corresponding to non-genomic hallmarks: senescence, disrupted proteostasis, deregulated nutrient sensing, altered intercellular communication, immune system dysfunction, mitochondrial dysfunction, and dysbiosis. Through Holmannova et al.’s (2024) overall descriptions for each non-genomic hallmark of aging, the research studies aimed to understand how they function and how they can be influenced and be beneficial in clinical practice, mainly in the treatment of age-related diseases such as cancer [21].

In the first paper, Kose et al. (2023) evaluated the concentrations of minichromosome maintenance-3 (MCM3) and envoplakin (EVPL) proteins in ThinPrep samples from patients with cervix neoplasia using a combination of targeted and untargeted proteomic approaches by the high-resolution tandem mass spectrometry system and verified if these markers may be used in population screening for cervix carcinoma [23]. Cervical cancer is the fourth most common cancer in women and is caused mainly by high-risk Human Papillomavirus (Hr-HPV) infection. In clinical settings, cytology combined with HPV screening is used to enhance test sensitivity and detect non-HPV-related cancers [24]. However, HPV testing is replacing cytology in population screening since it is used for risk assessment followed by triage based on genotyping or a combination of cytology and genotyping. Unfortunately, triage methods have moderate accuracy producing false-positive and false-negative results due to significant misdiagnosis associated with current methods (colposcopy, cytology, and histology), and thus there is need of other diagnostic parameters. In this context, Kose et al. (2023) collected 94 samples from Hr-HPV-positive patients among which 40 samples were “negative for intraepithelial lesion or malignancy” (NILM), 21 samples were “atypical squamous cells of undetermined significance” (ASC-US), and 33 samples were “low-grade squamous intraepithelial lesion and worse” (≥LSIL). Higher levels of MCM3 resulted in being mainly associated with precancerous stages of the cervix, confirming that this protein correlates strongly with cell division. On the other hand, the MCM3/EVPL ratio resulted in the ability to discriminate between non-dysplastic and dysplastic samples, evidencing that the decrease in EVPL levels highlighted that the cells lose their epithelial features and attachment to the basal membrane and start epithelial–mesenchymal transition, cellular migration, and invasion. Furthermore, the Receiver Operating Characteristic (ROC) curve plotted between NILM (defined by a Pap smear) and dysplastic (CIN1+ defined by colposcopy) groups evidenced that discrimination power of MCM3/EVPL had an area under the curve (AUC) of 0.80 versus an AUC of 0.74 in the case of cytology alone. Since large loop excision of the transformation zone (LLETZ) procedure is very accurate for the diagnosis of cervical intraepithelial neoplasia, Kose et al. (2023) applied this approach to a small number of samples and demonstrated that the MCM3/EVPL ratio had 100% agreement with LLETZ-histology confirmed samples. Therefore, the authors suggested that MCM3 and EVPL represent novel potential biomarkers for population-based cervical cancer screening [23].

In the second paper, using the flow cytometry procedure, Salvia et al. (2024) evaluated PD-L1 expression on circulating myeloid-derived suppressor cells (MDSCs) in non-small cell lung cancer (NSCLC) patients treated with anti-PD-1/PD-L1 immune checkpoint inhibitors (ICIs) [25]. These inhibitors have improved survival in NSCLC patients [26] even if a high percentage of patients still do not respond to ICIs. On the other hand, MDSCs are circulating cells that express PD-L1, infiltrate in the tumor microenvironment, and can induce immunosuppression [27]. The authors evaluated the circulating MDSC population and PDL1 expression in MDSCs in peripheral blood samples from 37 NSCLC patients before they started treatment with ICIs. Taking into account the mean fluorescence intensity (MFI) of stimulated cells normalized by the MFI of unstimulated cells, a PD-L1 index of MDSCs was defined to predict ICI escape in the patients. According to ROC curve analysis, the cutoff value of 5.88 resulted in the PD-L1 index value being able to predict progression-free survival in NSCLC patients with good sensitivity. Through Kaplan–Meier curves, the authors evidenced that the subgroup with the lower PD-L1 index significantly benefited from ICI treatment, having higher progression-free survival and overall survival compared to the subgroup with the higher PD-L1 index. However, no association was found between PD-L1 expression in tissues and the PD-L1 index identified in blood. Considering the follow-up of the NSCLC patients, it was found that in patients who did not progress, there were no significant differences in the PD-L1 index after ICI treatment, whereas in patients who progressed, there was a significant increase in the PD-L1 index, confirming that higher values of the PD-L1 index correlated with poor prognosis. Therefore, Salvia et al. (2024) concluded that the PD-L1 index may represent a novel quick tool to predict disease progression at baseline and the efficacy of anti-PD-1/PD-L1 treatment [25].

In the third article, de Falco et al. (2024) identified urinary para-hydroxyphenylacetic acid (u-pHPAA), a metabolite of tyrosine, as a potential biomarker for neuroendocrine neoplasms (NENs), comprising a heterogeneous group of neoplasms arising from the neuroendocrine system that most commonly occurs in the bronchopulmonary and gastropancreatic tract [28,29]. The management of NENs involves the evaluation of urinary 5-hydroxindolacetic acid (5-HIAA), serum neuro-specific enolase (s-NSE), and serum chromogranin A (s-CGA). Recently, higher levels of u-pHPAA was correlated to a worse outcome in NET patients [30]. In this context, de Falco et al. (2024) decided to evaluate the levels of s-CgA, s-NSE, u-5-HIAA, u-pHPAA, and tyrosine in blood or 24 h urine samples, collected at baseline (T0) and after 1 year of follow-up (T1), from 14 NEN patients. Their analyses demonstrated that at T0, s-CgA values were associated with death and the presence of metastasis whereas at T1, both s-CgA and u-5-HIAA levels were associated with death. Moreover, the values of the T1/T0 ratio for s-CgA continued to be associated with death as well for u-pHPAA. Additionally, tyrosine at T0 was correlated with death. Therefore, the authors suggested that the evaluation of u-PHPAA may be added to the commonly used biomarkers in order to improve NEN management [28].

This Special Issue collects articles that have suggested new and compelling biomarkers, although the limitation of all studies published in the literature on this topic is due to the fact that only a limited number of biomarkers have been successfully adopted in clinical practice. A significant limitation is the lack of standardized methodologies for sample collection, analysis, and interpretation of the results. This lack of uniformity can lead to inconsistencies in results across different studies and platforms, complicating the clinical translation of these technologies. Furthermore, the absence of standardized protocols makes it difficult to compare results obtained from different techniques, making it difficult to integrate the results into a cohesive diagnostic framework. Establishing common standards for liquid biopsy technologies and reporting practices is critical to ensure reproducibility and facilitate the reliable clinical application of these approaches.

## Figures and Tables

**Figure 1:**
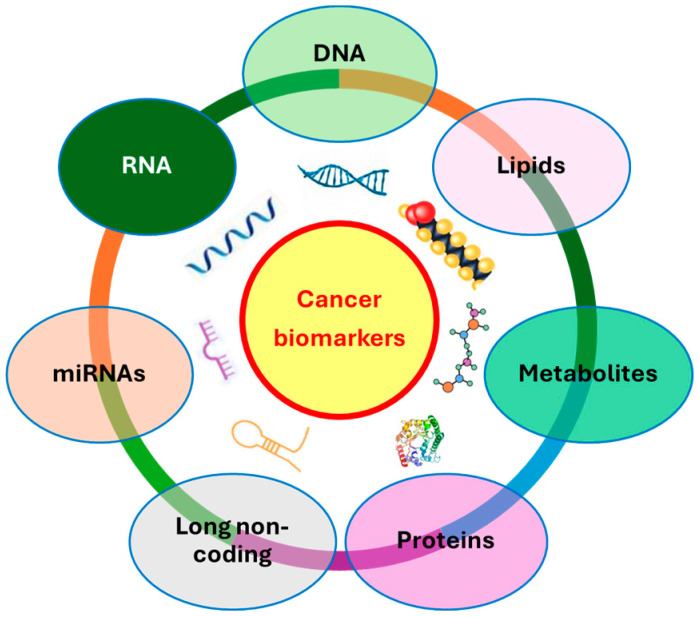
Schematic representation of potential cancer biomarkers.
